# Comparison of Risk of Target Organ Damage in Different Phenotypes of Arterial Stiffness and Central Aortic Blood Pressure

**DOI:** 10.3389/fcvm.2022.839875

**Published:** 2022-04-14

**Authors:** Yaya Bai, Qian Wang, Di Cheng, Yueliang Hu, Huijuan Chao, Alberto Avolio, Biwen Tang, Junli Zuo

**Affiliations:** ^1^Department of Geriatrics, Ruijin Hospital, Shanghai Jiao Tong University School of Medicine, Shanghai, China; ^2^Department of Biomedical Sciences, Faculty of Medicine, Health and Human Sciences, Macquarie (University) Medical School, Sydney, NSW, Australia

**Keywords:** arterial stiffness, carotid-femoral pulse wave velocity, central blood pressure, target organ damage, risk factors

## Abstract

**Objectives:**

The aim of this study was to explore the risk of target organ damage (TOD) in different groups based on carotid-femoral pulse wave velocity (cfPWV) and central aortic blood pressure (CBP) in different populations.

**Methods:**

The study cohort was divided into four groups according to the status of cfPWV and CBP [Group (*cfPWV/CBP*): high cfPWV and high CBP; Group (*cfPWV*): high cfPWV and normal CBP; Group (*CBP*): normal cfPWV and high CBP; Group (*control*): normal cfPWV and normal CBP]. TOD was determined by the assessment of carotid intima-media thickness (CIMT) abnormality, chronic kidney disease (CKD), microalbuminuria, and left ventricular hypertrophy (LVH).

**Results:**

A total of 1,280 patients (mean age 53.14 ± 12.76 years, 64.1% male patients) were recruited in this study. Regarding Group (*control*) as reference, LVH was significantly higher in Group (*cfPWV*) and Group (*CBP*) [OR 2.406, 95% CI (1.301–4.452), *P* < 0.05; OR 2.007, 95% CI (1.335–3.017), *P* < 0.05]; microalbuminuria was significantly higher in Group (*cfPWV/CBP*) and Group (*CBP*) [OR 3.219, 95% CI (1.630–6.359), *P* < 0.05; OR 3.156, 95% CI (1.961–5.079), *P* < 0.05]. With age stratified by 60 years, the risk of CKD was significantly higher in Group (*cfPWV/CBP*) [OR 4.019, 95% CI (1.439–11.229), *P* < 0.05].

**Conclusion:**

Different phenotypes based on the status of cfPWV and CBP were associated with different TOD. Individuals with both cfPWV and CBP elevated have a higher risk of microalbuminuria.

## Introduction

Various studies have demonstrated that arterial stiffness is associated with target organ damage (TOD), such as carotid intima-medium thickness (CIMT) abnormality, left ventricular hypertrophy (LVH), chronic kidney disease(CKD), and microalbuminuria (1–4). At present, the gold standard for evaluating arterial stiffness is carotid-femoral pulse wave velocity (cfPWV). cfPWV is one of the most frequently applied PWV measurements, which has a prognostic value not only for TOD but also for cardiovascular events.

Central aortic pressure is more closely associated with markers of vascular function and incidence of cardiovascular events compared with peripheral pressure (4). In 2019, the Taiwan Society of Cardiology (TSOC) and the Taiwan Society of Hypertension (THS) developed the consensus of clinical application of central blood pressure (CBP) in patients with hypertension, and CBP ≥ 130/90 mmHg was defined as hypertension. CBP is more predictive of TOD than peripheral blood pressure (PBP) (5). However, the potential clinical use of central aortic hemodynamic indices as markers of TOD has not been fully established (5, 6), and few studies have been conducted to elucidate the associations of TOD with cfPWV combined with CBP (1, 2). Based on this, we conducted this study aiming to risk stratify patients for TOD based on vascular risk parameters (cfPWV, CBP). Through this research, we hope to promote individualized diagnostic and therapeutic management of patients and to avoid overtreatment or insufficient treatment of patients by considering only a single biomarker (such as only cfPWV or CBP) in clinical practice.

## Methods

### Study Population

A total of 1,335 patients from Ruijin Hospital affiliated to Shanghai Jiao Tong University School of Medicine between December 2017 and August 2020 were included in this study. The inclusion criteria were health assessment population and age ≥18 years and ≤ 85 years. All patients gave written informed consent and accepted cfPWV and CBP examinations. Exclusion criteria were clinical or laboratory evidence confirming acute cardiovascular and cerebrovascular disease within the previous 3 months before enrollment or any life-threatening disease, including severe arrhythmias, such as atrial flutter, atrial fibrillation, ventricular premature beats, and ventricular tachycardia. Of the 1,335 patients, 11 cases and 44 cases were excluded due to missing data and data duplication, respectively. Finally, 1,280 patients were recruited in this study. Patients were divided into four groups according to the status of cfPWV and CBP: Group (*cfPWV/CBP*) (high cfPWV and high CBP), Group (*cfPWV*) (high cfPWV and normal CBP), Group (*CBP*) (normal cfPWV and high CBP), and Group (*control*) (normal cfPWV and normal CBP). cfPWV >10 m/s was defined as “high cfPWV,” and cfPWV ≤ 10 m/s was defined as “normal cfPWV” for cfPWV >10 m/s is a high-risk factor of asymptomatic hypertensive target organ damage (7). “High CBP” refers to central systolic blood pressure (cSBP) ≥130 mmHg and/or central diastolic blood pressure (cDBP) ≥90 mmHg, and “normal CBP” refers to cSBP < 130 mmHg and cDBP < 90 mmHg (8). Medical records, including age, sex, height, body mass index (BMI), smoking history (yes or no), antihypertensive drugs (yes or no), antilipidemic drugs (yes or no), heart rate (HR), and cfPWV, were collected. A sample of venous blood was drawn, and a sample of urine was collected after obtaining informed consent. Serum fasting glucose, hemoglobinA1c (HbA1c), creatinine (Cr), uric acid, triglyceride and total, low-density lipoprotein (LDL), and high-density lipoprotein (HDL) cholesterol were measured with standard methods on the venous blood sample; urinary albumin and creatinine were measured from the urine sample. Body mass index (BMI) was calculated as body weight in kilograms divided by the square of body height in meters; body surface area (BSA) was calculated using the formula: BSA (m^2^) = 0.0061 × body height (m) + 0.0128 × body weight (kg)−0.1529 (9). The study protocol was reviewed and approved by the Ethics Committee of Ruijin Hospital (Ethics No. 2011-30), Shanghai Jiao Tong University School of Medicine. All patients provided written informed consent.

### Indices of Central and Peripheral Hemodynamics

Radial waveforms and pulse wave analysis measurements were obtained by applanation tonometry using a high-fidelity SPT-304 micromanometer (Millar Instruments, Houston, TX) interfaced with a laptop computer. Central aortic pressure waveforms were derived from the radial waveforms with a validated transfer function using the SphygmoCor software, version 8.0 (AtCor Medical, Sydney, Australia) (10), and central hemodynamics indices, including central systolic blood pressure (cSBP), central diastolic blood pressure (cDBP), central mean arterial blood pressure (cMAP), central augmentation index (cAIx), and cAIx adjusted to heart rate of 75 bpm (beats per minute) (AIx@HR75), were generated. Radial waveforms were calibrated with the average of the peripheral systolic blood pressure (pSBP) and peripheral diastolic blood pressure (pDBP) measured 3 times at the left brachial artery with a validated Omron 705CP oscillometric device (Omron, Kyoto, Japan) (11), following at least 10 min of rest, and all measurements were performed in a quiet room with stable temperature with the subject in a supine position, avoiding smoking, caffeine, and exercise for 30 min (12). Peripheral mean arterial blood pressure (pMAP) was calculated for further study. Recordings were discarded when systolic or diastolic variability of consecutive waveforms exceeded 5% or when the amplitude of the pulse wave signal was <80 mV. All recordings met the manufacturer's quality control standards integrated into the software package.

### Carotid-Femoral Pulse Wave Velocity

Carotid-femoral pulse wave velocity was calculated as the measured distance from the suprasternal notch to the femoral artery subtracted by the distance from the suprasternal notch to the carotid artery and then divided by the pulse transit time. The fiducial point at the foot of the pulse for the measurement of transit time was determined by the intersecting tangent method, where a linear fit is performed on the late diastolic portion of the pulse and the early systolic rise of the subsequent pulse. The pulse transit time between the two arterial sites was determined as the difference between the R-wave of the electrocardiogram and the diastolic foot at the respective sites averaged over 10 consecutive heartbeats. Following the measurement of office blood pressure, carotid and femoral arterial waveforms at the patient's right side were obtained by applanation tonometry sequentially a short time apart. Patients fasted overnight, and no caffeine beverage or smoking was allowed within 3 h of the measurement. A single high-fidelity applanation tonometer SphygmoCor V8.0 device (AtCor Medical, Sydney, Australia) was used for the PWV measurements. cfPWV > 10 m/s was defined as “high cfPWV,” and cfPWV ≤ 10 m/s was defined as “normal cfPWV” in our study. We also estimated the cardio-ankle vascular index (CAVIo) using the same cfPWV value (13, 14).

### Target Organ Damage

#### Carotid Intima-Media Thickness

Carotid intima-media thickness was examined bilaterally using high-resolution Doppler ultrasound (HD11EX Ultrasound; Philips Medical Systems, Andover, MA, USA) with a broadband linear array transducer (multiple frequencies: 4–12 MHz). Three recordings were taken from the bulb origin (common carotid artery starting ~1.5 cm proximal to the carotid artery bulb) of both left and right carotid arteries during the diastolic portion of the cardiac cycle, and the average value of the three recordings was calculated for each side. Finally, CIMT was calculated as the average of the left CIMT and the right CIMT ([Left CIMT + Right CIMT]/2). CIMT > 1.3 mm was diagnosed with carotid plaque. CIMT abnormality was diagnosed as CIMT ≥0.9 mm and/or the presence of carotid plaque.

#### Left Ventricular Hypertrophy

Cardiac dimensions were measured based on a standardized reading protocol, and all indices were evaluated by an experienced sonographer or cardiologist. Cardiac dimensions were quantified using digital images and the leading-edge technique as recommended by the American Society of Echocardiography. M-mode echocardiography was used to obtain linear measurements of the left ventricular (LV) cavity [LV end-diastolic diameter and LV end-systolic diameter (LVESD), interventricular septum thickness (IVST), and posterior wall thickness (PWT)]. Left ventricular mass (LVM) was calculated according to the American Society of Echocardiography guidelines (15). LVM was calculated with the formula: LVM (g) = 0.8 × [1.04 × [(IVST + PWT + LVDD)^3^-(LVDD)^3^] + 0.6. Body surface area (BSA) was calculated using the formula: BSA (m^2^) = 0.0061 × body height (m) + 0.0128 × body weight (kg)−0.1529. Left ventricular mass index (LVMI) = LVM/BSA (g/m^2^). We defined LVH as LVMI >95 g/m^2^ and >115 g/m^2^ for women and men, respectively.

#### Renal Abnormalities

Urinary albumin-creatinine ratio (ACR) was used to screen patients with urinary albuminuria. ACR was measured from spot morning urine samples obtained from participants. ACR values have been shown to identify kidney disease that occurs as a complication with hypertension. Abnormal albuminuria was defined as a urine ACR >3.5 mg/mmol in female patients and >2.5 mg/mmol in male patients. The definition and the diagnostic criteria for chronic kidney disease were proposed in the K/DOQI guidelines (16): estimated glomerular filtration rate (eGFR <60 ml/min/1.73 m^2^) calculated by the MDRD formula: eGFR (ml/min/1.73 m^2^) = 175 × Cr (mg/dl)^−1.234^ × age (years old)^−0.179^ × 0.79 (if female patient).

### Statistical Analysis

All analyses were performed using SPSS 24.0 for Windows (SPSS Inc, Chicago, IL, USA). A two-sided *P* < 0.05 was considered statistically significant. Quantitative and qualitative parameters were presented as mean ± standard deviation and numbers with the percentage in parentheses, compared among groups by one-way ANOVA and chi-squared test, respectively. Pearson's correlation analysis was applied to investigate the correlation of central and peripheral hemodynamic indices with TOD. Furthermore, the relative odds ratios by multivariate stepwise linear or logistic regressions analysis [forward likelihood ratio (LR)] were conducted to compare the associations of risk factors with TOD among different groups after adjusting for age, sex, BMI, height, smoking history, antihypertensive drugs (yes or no), HDL-c, LDL-c, FBG, HR, and pMAP. Only variables staying in the final model were presented.

## Results

### Baseline Characteristics of the Studied Population

A total of 1,280 patients (mean age 53.14 ± 12.76 years, 64.06% male patients) were recruited in this study; 30.8% of patients were taking antihypertensive drugs; and 16.8% of patients had a smoking history. ACR was skewed so Log ACR was used for the logistic regression. Mean values of CIMT, LVMI, eGFR, and LogACR were significantly different in the four groups (*P* < 0.05). The percentages of TOD were significantly different among Group (*cfPWV/CBP*) to Group (*control*) (*P* < 0.001). Age, BMI, triglycerides, cholesterol, FBG, HbA1c, HR, cfPWV, and CAVIo were all significantly different among groups (*P* < 0.05). As for central and peripheral blood hemodynamic indices, patients in Group (*cfPWV/CBP*) had significantly higher levels of pSBP and cSBP than the other three groups (*P* < 0.05). Patients in Group (*CBP*) had higher levels of pDBP, cDBP, and cAIx than Group (*cfPWV*) and Group (*control*) (*P* < 0.05) (Table 1).

**Table 1:** Baseline characteristics of the studied population.

**Variance**	**Overall**	**Group (*cfPWV/CBP*)**	**Group (*cfPWV*)**	**Group (*CBP*)**	**Group (*control*)**	***P value***
	***N* = 1,280**	***N* = 106**	***N* = 92**	***N* = 286**	***N* = 771**	
Age (years)	53.14 ± 12.76	61.65 ± 11.43^bcd^	66.88 ± 10.62^acd^	52.04 ± 11.46^ab^	50.75 ± 11.93 ^ab^	**<0.001**
Sex						
Male	820	68 (64.2%)	63 (68.5%)	182 (63.6%)	493 (63.9%)	
Female	460	38 (35.8%)	29 (31.5%)	104 (36.4%)	278 (36.1%)	0.851
Height (cm)	167.38 ± 8.24	167.09 ± 8.39	166.36 ± 7.88	166.78 ± 8.68	167.80 ± 7.98	0.164
BMI (Kg/m2)	25.35 ± 3.96	25.58 ± 4.05	25.13 ± 3.44^c^	26.34 ± 3.91^bd^	24.93 ± 3.86^c^	**<0.001**
Waist-to-hip ratio	0.93 ± 0.08	0.96 ± 0.07^cd^	0.98 ± 0.09^cd^	0.94 ± 0.08^ab^	0.93 ± 0.08^ab^	**<0.001**
**Cardiovascular risk factors**						
Smoking history, *n* (%)	215 (16.8%)	17/106 (16.0%)	14/92 (15.2%)	45/286 (15.7%)	139/771 (18.0%)	0.763
Antihypertensive drugs, *n* (%)	394 (30.8%)	44/106 (41.5%)	32/92 (34.8%)	91/286 (31.8%)	224/771 (29.1%)	0.057
Serum uric acid (umol/L)	365.01 ± 96.34	378.48 ± 91.99	364.61 ± 91.54	373.61 ± 99.86	360.33 ± 96.04	0.118
Triglycerides (mmol/L)	1.93 ± 1.61	2.02 ± 1.21	1.80 ± 1.23	2.18 ± 2.16^d^	1.85 ± 1.46^c^	**0.006**
Total cholesterol (mmol/L)	4.80 ± 1.07	4.85 ± 1.05^b^	4.49 ± 0.97^acd^	4.90 ± 1.22^b^	4.80 ± 1.03^b^	**0.027**
HDL-c (mmo/L)	1.15 ± 0.35	1.11 ± 0.23	1.17 ± 0.49	1.11 ± 0.31^d^	1.16 ± 0.36^c^	0.104
LDL-c (mmol/L)	3.13 ± 0.80	3.15 ± 0.80^b^	2.90 ± 0.74^acd^	3.16 ± 0.84^b^	3.14 ± 0.80^b^	0.053
FBG (mmol/L)	5.79 ± 1.77	6.44 ± 2.31^cd^	6.44 ± 2.54^cd^	5.81 ± 1.71^ab^	5.61 ± 1.56^ab^	**<0.001**
HbA1c (%)	6.16 ± 1.22	6.71 ± 1.66^cd^	6.60 ± 1.16^cd^	6.10 ± 1.21^ab^	6.03 ± 1.11^ab^	**<0.001**
Heart rate (beat/min)	69.31 ± 10.52	71.92 ± 9.99 ^cd^	71.57 ± 9.92 ^cd^	68.63 ± 10.98 ^ab^	66.84 ± 10.21 ^ab^	**0.003**
cf-PWV (m/s)	8.24 ± 2.02	11.94 ± 1.35 ^cd^	11.77 ± 1.70 ^cd^	8.30 ± 1.02^abd^	7.30 ± 1.19^abc^	**<0.001**
**Peripheral blood pressure**						
pSBP (mmHg)	130.73 ± 18.63	155.94 ± 14.70^bcd^	130.86 ± 9.83^acd^	149.16 ± 11.24^abd^	120.30 ± 12.29^abc^	**<0.001**
pDBP (mmHg)	76.71 ± 11.97	86.82 ± 11.11^bd^	71.95 ± 8.03^ac^	88.37 ± 10.14^bd^	71.54 ± 8.76^ac^	**<0.001**
p-MAP (mmHg)	94.72 ± 13.10	109.86 ± 9.69^bd^	91.58 ± 7.22^acd^	108.64 ± 8.32^bd^	87.80 ± 9.00^abc^	**<0.001**
**Central blood pressure**						
cAIx	28.71 ± 13.17	30.87 ± 9.85^bd^	25.18 ± 12.47^ac^	32.66 ± 11.45^bd^	27.53 ± 13.81^ac^	**<0.001**
cAIx@HR75	25.25 ± 11.86	29.34 ± 8.66 ^bd^	23.39 ± 12.28 ^ac^	28.75 ± 9.32 ^bd^	23.71 ± 12.58 ^ac^	**<0.001**
cSBP (mmHg)	119.69 ± 17.95	143.43 ± 13.82^bcd^	117.49 ± 8.52^acd^	138.73 ± 11.04^abd^	109.58 ± 11.16^abc^	**<0.001**
cDBP (mmHg)	77.82 ± 12.11	88.25 ± 11.26^bd^	72.92 ± 8.21^ac^	89.64 ± 10.14^bd^	72.55 ± 8.82^ac^	**<0.001**
c-MAP (mmHg)	95.53 ± 13.95	111.46 ± 10.80^bd^	91.41 ± 7.93^acd^	110.54 ± 8.77^bd^	88.24 ± 9.45^abc^	**<0.001**
CAVIo	2.260 ± 1.090	3.729 ± 1.689 ^bcd^	4.527 ± 1.546 ^acd^	1.816 ± 0.531 ^ab^	1.952 ± 0.532 ^ab^	**<0.001**
**Target organ parameters**						
eGFR [mL/(min 1.73 m2)]	90.28 ± 16.95	81.89 ± 20.73^bcd^	86.91 ± 19.70^acd^	90.50 ± 16.51^a^	91.76 ± 15.55^ab^	**<0.001**
LVMI (g/m2)	103.60 ± 26.59	118.23 ± 31.11 ^cd^	114.57 ± 28.14 ^d^	110.21 ± 25.84^ad^	98.39 ± 24.12^abc^	**<0.001**
CIMT (mm)	0.74 ± 0.14	0.81 ± 0.18 ^cd^	0.80 ± 0.15^cd^	0.74 ± 0.14^ab^	0.73 ± 0.13^ab^	**<0.001**
Log ACR (mg/mmol)	0.39 ± 0.37	0.52 ± 0.43^d^	0.54 ± 0.56 ^d^	0.44 ± 0.44 ^d^	0.34 ± 0.29 ^abc^	**0.020**
**Target organ damage**						
LVH (*n*, %)	308/853 (36.1%)	35/65 (53.8%)^d^	41/68 (60.3%)^cd^	70/156 (44.9%)^bd^	153/548 (27.9%)^acb^	**<0.001**
CIMT abnormality (*n*, %)	394/804 (49.0%)	50/71 (70.4%)^cd^	51/66 (77.3%)^cd^	75/162 (46.3%)^ab^	211/493 (42.8%)^ab^	**<0.001**
ACR abnormality (*n*, %)	134/766 (17.5%)	19/49 (38.8%)^bd^	11/60 (18.3%)^ac^	43/134 (32.1%)^bd^	56/512 (10.9%)^ac^	**<0.001**
CKD (*n*, %)	37/1,213 (3.1%)	11/102 (10.8%)^cd^	4/90 (4.4%)	8/270 (3.0%)^a^	13/730 (1.8%)^a^	**<0.001**

*Groups by cfPWV and CBP status: Group (cfPWV/CBP): high cfPWV and high CBP; Group (cfPWV): high cfPWV and normal CBP; Group (CBP): normal cfPWV and high CBP; Group (control) : normal cfPWV and normal CBP*.

*Data are mean ± SD or as stated. P-value: independent t-test ANOVA for numeric variables and chi-square test for categorical variables. HDL-c, high-density lipoprotein c; LDL-c, low-density lipoprotein c; FBG, fasting blood glucose; pSBP, peripheral systolic blood pressure; pDBP, peripheral diastolic blood pressure; p-MAP, peripheral mean arterial pressure; cCSBP, central systolic blood pressure; cDBP, central diastolic blood pressure; c-MAP, central mean arterial pressure; cAIx, central augmentation index; cAIx@HR75, cAIx adjusted to the heart rate of 75 bpm. cfPWV, carotid-femoral pulse wave velocity. CAVIo, cardio-ankle vascular index. eGFR, estimated glomerular filtration rate; LVMI, left ventricular myopathy index; CIMT, carotid intima-media thickness; ACR, albumin–creatinine ratio; LVH, left ventricular hypertrophy; CKD, chronic kidney disease*.

*Superscripts a, b, c, and d stand for significant difference with Group (cfPWV/CBP), Group (cfPWV), Group (CBP), and Group (control), respectively*.

*The bold values represent the values that are statistically significant (P < 0.05)*.

### Correlation Between Central or Peripheral Hemodynamic Indices and TOD

**Figure 1:**
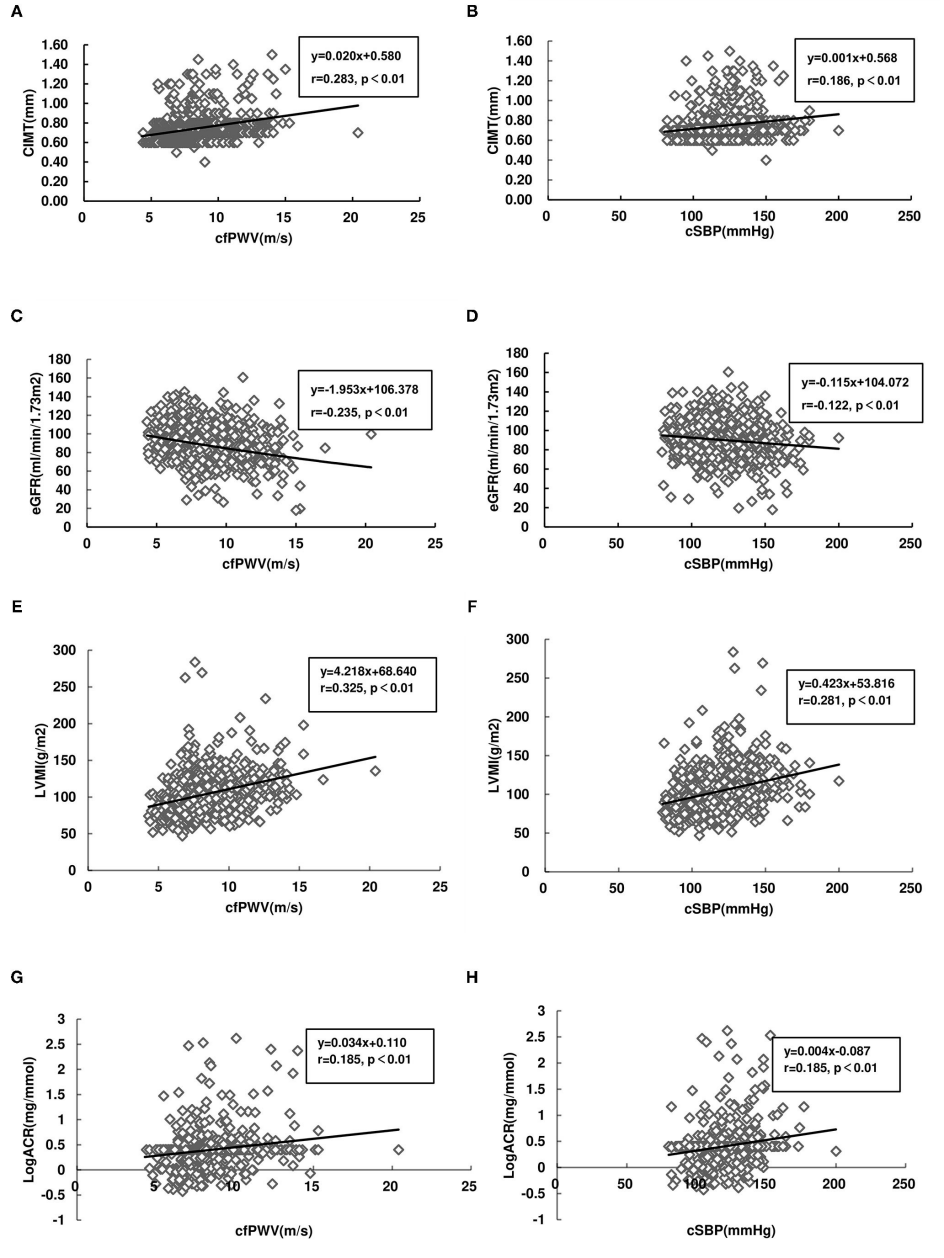
Correlations between target organ damage (TOD) and carotid-femoral pulse wave velocity (cfPWV), as well as central systolic blood pressure (cSBP), carotid intima-media thickness (CIMT), and cfPWV **(A)**; CIMT and cSBP **(B)**; estimated glomerular filtration rate (eGFR) and cfPWV **(C)**; eGFR and cSBP **(D)**; left ventricular mass index (LVMI) and cfPWV **(E)**; LVMI and cSBP **(F)**; Log (urinary albumin-creatinine ratio, ACR) (LogACR) and cfPWV **(G)**; and LogACR and cSBP **(H)**. Linear regression lines for (x) and (y) variables are shown with correlation coefficients and *P*-values.

In the overall studied population, cfPWV and cSBP were positively correlated with CIMT (*r* = 0.283, *P* < 0.01; *r* = 0.186, *P* < 0.01) (Figures 1A,B). cfPWV and cSBP were negatively correlated with eGFR (*r* = −0.235, *P* < 0.01; *r* = −0.122, *P* < 0.01) (Figures 1C,D). Both cfPWV and cSBP were positively correlated with LVMI(*r* = 0.325, *P* < 0.01; *r* = 0.281, *P* < 0.01) (Figures 1E,F), and LogACR (*r* = 0.185, *P* < 0.01; *r* = 0.185, *P* < 0.01) (Figures 1G,H).

### Multivariate Stepwise Linear and Logistic Regression Analysis of the Relationship Between Arterial Stiffness Indices or CBP With the Presence of TOD

In the overall study population, according to multiple stepwise linear regression analysis, cSBP was positively correlated with CIMT (β = 0.095, *P* = 0.009), cSBP and cfPWV were positively correlated with LVMI (β = 0.105*, P* = 0.008; β = 0.137, *P* = 0.003), cDBP was negatively correlated with eGFR (β = −0.103*, P* = 0.001), whereas cAIx@HR75 was positively correlated with eGFR (β = 0.080*, P* = 0.017) and cSBP was positively correlated with LogACR (β = 0.186*, P < * 0.001) after adjusting for age, sex, height, BMI, smoking history, antihypertensive drugs (yes or no), HDL-c, LDL-c, FBG, pMAP, and HR. Age was the main independent influence factor of CIMT, eGFR, and LVMI, while cSBP affected LogACR more compared with age and FBG (Table 2).

**Table 2:** Multiple stepwise linear regression of risk factors of target organ damage in the overall studied population.

**Variance**		**B**	**SE**	**β**	***P* value**	**95% CI**	**VIF**
CIMT	Age	0.004	0.000	0.402	**<0.001**	0.040–0.050	1.060
	Sex	−0.028	0.011	−0.093	**0.012**	−0.049–0.006	1.109
	HDL-c	−0.039	0.017	−0.083	**0.025**	−0.074–0.005	1.100
	cSBP	0.001	0.000	0.095	**0.009**	0.000–0.001	1.061
LVMI	Age	0.404	0.086	0.194	**<0.001**	0.235–0.573	1.545
	Sex	−8.411	2.045	−0.149	**<0.001**	−12.426–4.396	1.183
	BMI	0.568	0.253	0.085	**0.025**	0.072–1.064	1.300
	Antihypertensive drugs (yes or no)	5.106	1.885	0.097	**0.007**	1.406–8.807	1.158
	HDL-c	−5.964	2.719	−0.076	**0.029**	−11.303–0.626	1.097
	HR	−0.373	0.093	−0.137	**<0.001**	−0.557–0.19	1.067
	cSBP	0.156	0.058	0.105	**0.008**	0.042–0.271	1.396
	cf-PWV	1.775	0.589	0.137	**0.003**	0.618–2.931	1.876
eGFR	Age	−0.555	0.042	−0.428	**<0.001**	−0.638–0.473	1.246
	Sex	−5.460	1.408	−0.161	**<0.001**	−8.224–2.697	2.055
	Height	−0.176	0.083	−0.090	**0.034**	−0.338–0.014	2.148
	FBG	0.792	0.273	0.087	**0.004**	0.257–1.328	1.074
	LDL-c	−2.326	0.584	−0.117	**<0.001**	−3.473–1.179	1.034
	HR	0.174	0.052	0.110	**0.001**	0.073–0.276	1.279
	cDBP	−0.136	0.041	−0.103	**0.001**	−0.216–0.055	1.176
	cAIx@HR75	0.100	0.042	0.080	**0.017**	0.018–0.183	1.349
Log ACR	Age	0.003	0.001	0.110	**0.010**	0.001–0.005	1.060
	FBG	0.017	0.008	0.094	**0.028**	0.002–0.033	1.054
	cSBP	0.004	0.001	0.186	**<0.001**	0.002–0.006	1.023

*All variables adjusted for age, sex (male or female), height, BMI, body mass index, smoking history, antihypertensive drugs (yes or no), HDL-c, high-density lipoprotein cholesterol; LDL-c, low-density lipoprotein cholesterol; FBG, fasting blood glucose; pMAP, peripheral mean arterial pressure; HR, heart rate beats per minute; VIF, variance inflation factor*.

*The bold values represent the values that are statistically significant (P < 0.05)*.

According to multiple stepwise logistic regression, we found that cAIx@HR75 was significantly associated with LVH [OR 0.959, 95% CI (0.936–0.983), *P* = 0.001], cfPWV was significantly associated with CKD [OR 1.303, 95% CI (1.096–1.550), *P* = 0.003], and cSBP was significantly associated with ACR abnormality [OR 1.034, 95% CI (1.020–1.048), *P* < 0.001] after adjusting for age, sex, height, BMI, smoking history, antihypertensive drugs (yes or no), HDL-c, LDL-c, FBG, pMAP, and HR (Table 3).

**Table 3:** Multiple stepwise logistic regression of risk factors of target organ damage in the overall studied population.

**Variance**		**β**	**SE**	**Exp β**	**95% CI**	***P* value**
CIMT abnormality	Age	0.087	0.008	1.091	1.073–1.108	**<0.001**
	Sex	−0.636	0.183	0.530	0.370–0.758	**0.001**
	Antihypertensive drugs (yes or no)	0.548	0.167	1.730	1.247–2.401	**0.001**
	FBG	0.121	0.054	1.129	1.015–1.255	**0.025**
LVH	Age	0.052	0.008	1.054	1.038–1.070	**<0.001**
	Sex	0.758	0.198	2.134	1.448–3.145	**<0.001**
	BMI	0.065	0.024	1.067	1.017–1.119	**0.008**
	cSBP	0.013	0.007	1.013	1.000–1.026	0.053
	cAIx@HR75	−0.041	0.012	0.959	0.936–0.983	**0.001**
CKD	Age	0.049	0.021	1.050	1.008–1.093	**0.018**
	cf-PWV	0.265	0.088	1.303	1.096–1.550	**0.003**
ACR abnormality	Antihypertensive drugs (yes or no)	0.593	0.233	1.810	1.145–2.859	**0.011**
	FBG	0.147	0.052	1.158	1.046–1.282	**0.005**
	cSBP	0.033	0.007	1.034	1.020–1.048	**<0.001**

*All variables adjusted for age, sex (male or female), height, BMI, body mass index, smoking history, antihypertensive drugs (yes or no), HDL-c, high-density lipoprotein cholesterol; LDL-c, low-density lipoprotein cholesterol; FBG, fasting blood glucose; pMAP, peripheral mean arterial pressure; HR, heart rate beats per minute; VIF, variance inflation factor*.

*The bold values represent the values that are statistically significant (P < 0.05)*.

### Comparing the Risk of TOD in Different Groups Based on the Status of cfPWV and CBP by Multiple Stepwise Logistic Regression Analysis

**Figure 2:**
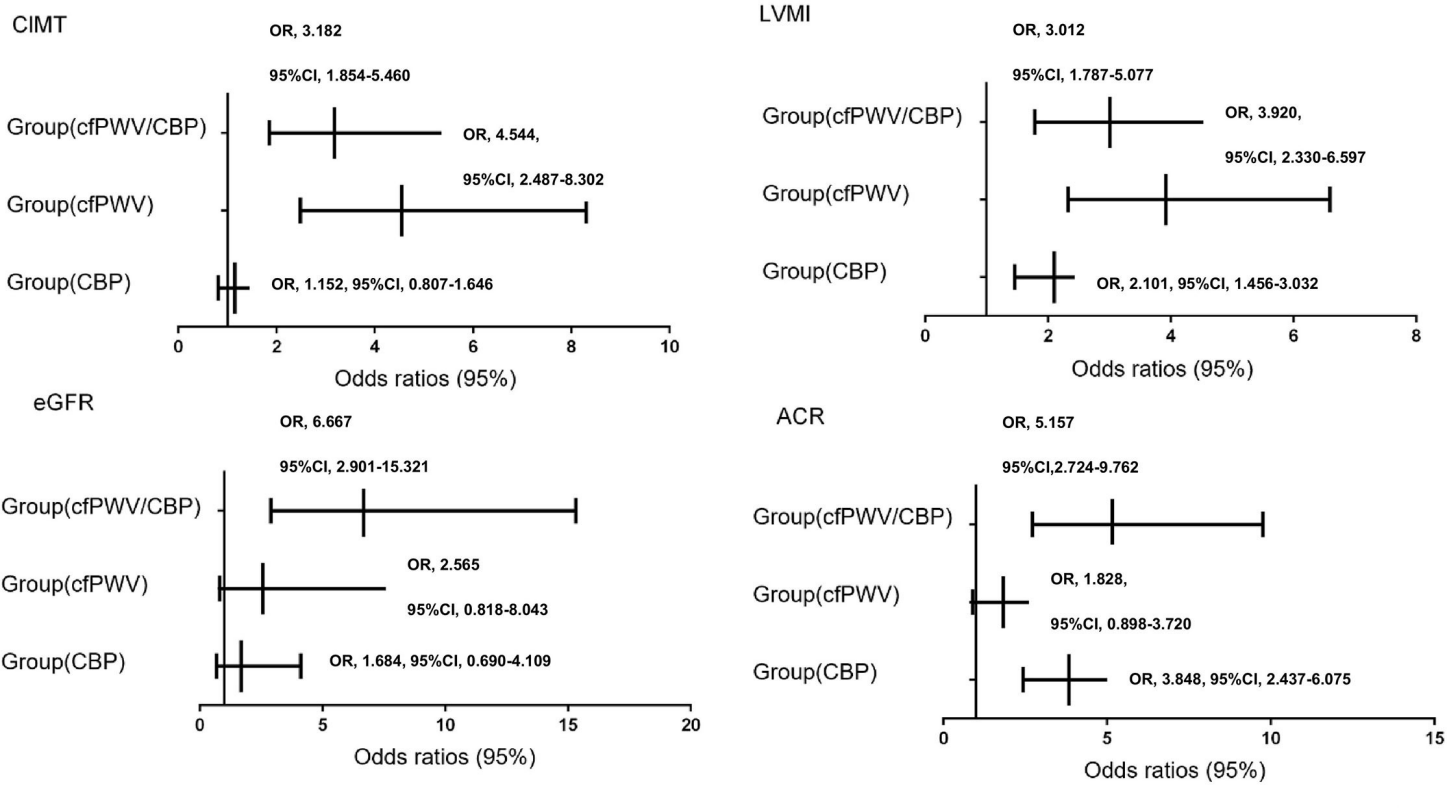
Multiple stepwise logistic regression of target organ damage among groups by cf-PWV and CBP without adjusted factors. Groups by cfPWV and CBP status: Group (*cfPWV/CBP*): high cfPWV and high CBP; Group (*cfPWV*): high cfPWV and normal CBP; Group (*CBP*): normal cfPWV and high CBP; Group (*control*): normal cfPWV and normal CBP.

Without adjusting for covariates, regarding Group (*control*) as reference, CIMT abnormality was significantly higher in Group (*cfPWV/CBP*) and Group (*cfPWV*) [OR 3.182, 95% CI (1.854–5.460), *P* < 0.01; OR 4.544, 95% CI (2.487–8.302), *P* < 0.01]. LVH was significantly higher in Group (*cfPWV/CBP*), Group (*cfPWV*), and Group (*CBP*) [OR 1 3.012, 95% CI (1.787–5.077), *P* < 0.01; OR 2 3.920, 95% CI (2.330–6.597), *P* < 0.01; OR 3 2.101, 95% CI (1.456–3.032), *P* < 0.01]. CKD had a significantly higher prevalence in Group (*cfPWV/CBP*) [OR 6.667, 95% CI (2.901–15.321), *P* < 0.01]. ACR abnormality was significantly higher in Group (*cfPWV/CBP*) and Group (*CBP*) [OR 5.157, 95% CI (2.724–9.762), *P* < 0.01; OR 3.848, 95% CI (2.437–6.075), *P* < 0.01] (Figure 2).

After adjusting for covariates, such as age, sex, height, BMI, smoking history, antihypertensive drugs (yes or no), HDL-c, LDL-c, FBG, pMAP, and HR, we found that LVH was significantly higher in Group (*cfPWV*) and Group (*CBP*) [OR 2.406, 95% CI (1.301–4.452), *P* = 0.005; OR 2.007, 95% CI (1.335–3.017), *P* = 0.001]. ACR abnormality was significantly higher in Group (*cfPWV/CBP*) and Group (*CBP*) [OR 3.219, 95% CI (1.630–6.359), *P* = 0.001; OR 3.156, 95 % CI (1.961–5.079), *P* < 0.001] (Table 4). As age was the main susceptibility factor of CKD and CIMT abnormality, we performed the subgroup analysis of eGFR abnormality and CIMT abnormality in different groups by stratifying age by 60 years according to the international standards for the definition of elderly at 60 years (17). Furthermore, we found that CKD was significantly higher in Group (*cfPWV/CBP*) [OR 4.019, 95% CI(1.439–11.229), *P* = 0.008] (Table 5), and CIMT abnormality was significantly lower in Group (*CBP*) [OR 0.466, 95% CI (0.231–0.941), *P* = 0.033] (Table 6).

**Table 4:** Multiple stepwise logistic regression of target organ damage in different groups.

**Variance**		**β**	**SE**	**Exp β**	**95% CI**	***P* value**
CIMT abnormality	Age	0.085	0.008	1.088	1.071–1.106	**<0.001**
	Sex	−0.714	0.188	0.490	0.339–0.709	**<0.001**
	BMI	−0.050	0.024	0.951	0.907–0.997	**0.037**
	Antihypertensive drugs (yes or no)	0.644	0.174	1.905	1.354–2.680	**<0.001**
	FBG	0.128	0.056	1.137	1.019–1.267	**0.021**
LVH	Age	0.047	0.008	1.048	1.032–1.064	**<0.001**
	Sex	0.949	0.187	2.582	1.789–3.728	**<0.001**
	BMI	0.050	0.023	1.051	1.004–1.100	**0.033**
	Antihypertensive drugs (yes or no)	0.362	0.171	1.437	1.028–2.007	**0.034**
	HDL-c	−0.719	0.324	0.487	0.258–0.920	**0.027**
	HR	−0.023	0.009	0.978	0.961–0.995	**0.010**
	Group (*control*) (Ref)					**0.001**
	Group (*cfPWV/CBP*)	0.566	0.306	1.762	0.967–3.209	0.064
	Group (*cfPWV*)	0.878	0.314	2.406	1.301–4.452	**0.005**
	Group (*CBP*)	0.696	0.208	2.007	1.335–3.017	**0.001**
CKD	Age	0.072	0.016	1.074	1.040–1.109	**<0.001**
ACR abnormality	Antihypertensive drugs (yes or no)	0.826	0.220	2.284	1.485–3.513	**<0.001**
	FBG	0.147	0.049	1.159	1.052–1.276	**0.003**
	Group (*control*) (Ref)					**<0.001**
	Group (*cfPWV/CBP*)	1.169	0.347	3.219	1.630–6.359	**0.001**
	Group (*cfPWV*)	0.258	0.388	1.294	0.605–2.767	0.507
	Group (*CBP*)	1.149	0.243	3.156	1.961–5.079	**<0.001**

*All variables adjusted for age, sex (male or female), height, BMI, body mass index, smoking history, antihypertensive drugs (yes or no), HDL-c, high-density lipoprotein cholesterol; LDL-c, low-density lipoprotein cholesterol; FBG, fasting blood glucose; pMAP, peripheral mean arterial pressure; HR, heart rate beats per minute; VIF, variance inflation factor*.

*The bold values represent the values that are statistically significant (P < 0.05)*.

**Table 5:** Subgroup analysis of eGFR abnormality adjusting for age in different groups.

**Variance**		**β**	**SE**	**Exp β**	**95% CI**	***P* value**
Model 1	Group (*control*) (Ref)					**0.002**
	Group (*cfPWV/CBP*)	1.769	0.492	5.867	2.236–15.393	**<0.001**
	Group (*cfPWV*)	0.851	0.672	2.342	0.627–8.741	0.205
	Group (*CBP*)	0.051	0.598	1.052	0.326–3.397	0.932
Model 2	Age (≥60 years)	0.943	0.456	2.567	1.049–6.279	**0.039**
	Group (*control*) (Ref)					**0.042**
	Group (*cfPWV/CBP*)	1.391	0.524	4.019	1.439–11.229	**0.008**
	Group (*cfPWV*)	0.350	0.709	1.418	0.353–5.695	0.622
	Group (*CBP*)	−0.007	0.600	0.993	0.306–3.219	0.990

*Model 1 adjusted for sex (male or female), height, BMI, body mass index, smoking history, antihypertensive drugs (yes or no), high-density lipoprotein cholesterol HDL-c, high-density lipoprotein cholesterol; LDL-c, low-density lipoprotein cholesterol; HbA1c, hemoglobinA1c; HR, heart rate; p-MAP,peripheral mean arterial pressure, and groups*.

*Model 2 adjusted for sex, height, BMI, smoking history, antihypertensive drugs (yes or no), HDL-c, LDL-c, HbA1c, heart rate, p-MAP, groups, and age*.

*The bold values represent the values that are statistically significant (P < 0.05)*.

**Table 6:** Subgroup analysis of CIMT abnormalities adjusting for age among different groups by cfPWV and CBP.

	**Variance**	**β**	**SE**	**Exp β**	**95% CI**	***P* value**
Model 1	Antihypertensive drugs (yes or no)	1.340	0.262	3.817	2.285–6.377	**<0.001**
Model 2	Age (≥60 years)	1.345	0.301	3.836	2.125–6.925	**<0.001**
	Antihypertensive drugs (yes or no)	1.317	0.273	3.731	2.186–6.366	**<0.001**
Model 3	Age (≥60 years)	1.319	0.306	3.739	2.054–6.808	**<0.001**
	Antihypertensive drugs (yes or no)	1.121	0.282	3.068	1.764–5.336	**<0.001**
	Lipid-lowering drugs (yes or no)	0.726	0.265	2.066	1.229–3.474	**0.006**
Model 4	Age (≥60 years)	1.161	0.327	3.192	1.682–6.058	**<0.001**
	Antihypertensive drugs (yes or no)	1.309	0.302	3.703	2.048–6.693	**<0.001**
	Lipid-lowering drugs (yes or no)	0.671	0.272	1.956	1.149–3.330	**0.013**
	Group (*control*) (Ref)					**0.043**
	Group (*cfPWV/CBP*)	−0.163	0.504	0.850	0.317–2.281	0.747
	Group (*cfPWV*)	2.008	1.117	7.447	0.835–66.442	0.072
	Group (*CBP*)	−0.763	0.358	0.466	0.231–0.941	0.033

*Model 1 adjusted for sex (male or female), height, BMI, body mass index, smoking history, heart rate, peripheral mean arterial pressure (p-MAP), glucose-lowering drugs (yes or no), and antihypertensive drugs (yes or no)*.

*Model 2 adjusted for sex, height, BMI, smoking history, heart rate, p-MAP, glucose-lowering drugs (yes or no), antihypertensive drugs (yes or no), and age*.

*Model 3 adjusted for sex, height, BMI, smoking history, heart rate, p-MAP, glucose-lowering drugs (yes or no), antihypertensive drugs (yes or no), age, and lipid-lowering drugs (yes or no)*.

*Model 4 adjusted for sex, height, BMI, smoking history, heart rate, p-MAP, glucose-lowering drugs (yes or no), antihypertensive drugs (yes or no), age, lipid-lowering drugs (yes or no), and groups*.

*The bold values represent the values that are statistically significant (P < 0.05)*.

## Discussion

Through multiple stepwise linear or logistic regression analysis, we found that CBP parameters and cfPWV were significantly correlated with cardiovascular and renal damage indexes. By group comparison, Group (*cfPWV/CBP*), individuals with high cf-PWV and high CBP, had an increased risk of microalbuminuria; Group (*cfPWV*), individuals with high cf-PWV and low CBP, had an increased risk of LVH, while Group (*CBP*) of low cf-PWV and high CBP had an increased risk of both microalbuminuria and LVH, with OR values lower than the first two groups. The prevalence of CKD and CIMT abnormality in the three groups was not significantly different from that in the control group.

cf-PWV, identified by meta-analyses as a predictor of future CVD events and all-cause mortality independent of blood pressure (6, 18), has also been associated with a decreased renal function (19–21). On the contrary, a number of studies have shown that central aortic blood pressure could better reflect the load on the heart and central vasculature (22, 23) and is associated with cardiovascular outcomes and mortality independent of peripheral brachial arterial pressure (24). In this study, CBP parameters and cf-PWV were found to be significantly correlated with cardiovascular and renal damage indexes, consistent with previous studies.

Although there is a strong relationship between blood pressure and vascular stiffness, the correlation between various blood pressure parameters and cf-PWV could be different (25) and varies according to the age range of the population studied (26–28), not to mention that cf-PWV is influenced by many other factors, such as gender, heart rate, salt intake, or genetic factors. Therefore, it is not surprising that CBP and cf-PWV are partly inconsistent in the study population. We found discordant CBP and cf-PWV status in 378 of 1,280 participants (29.5%): 92 with normal CBP and high cf-PWV and 286 with high CBP and normal cf-PWV. We questioned if the population was divided into different subgroups based on cf-PWV and CBP, whether the groups would show inconsistency in screening TOD?

A small number of studies showed apparent inconsistencies between CBP and cf-PWV. In a sub-study of the Framingham Heart Study, a similar grouping method was used to investigate the relative predictive value of central pulse pressure and cf-PWV for LVH and CVD, and it was concluded that in the community-dwelling middle-aged population, disagreement between pulse pressure and cf-PWV is common, with the highest prevalence of LVH and highest risk of CVD in people with both being elevated (29). However, in our study, regarding Group (*control*) as a reference, LVH was significantly higher in Group (*cfPWV/CBP*), Group (*cfPWV*), and Group (*CBP*) without adjusting covariates. After adjusting for covariates, Group (*cfPWV/CBP*) was not associated with LVH [OR = 1.762, *P* = 0.064]. In accordance with “Framingham Heart Study” (29), we additionally adjusted for height and pMAP in multiple logistic regression analysis. By screening covariates, we found that age was the main factor. Hence, this result illustrates the significant role of adjusting potential confounding variables that influence the risk factors.

In our study, individuals with only cf-PWV elevated had the highest risk of LVH, while the risk of microalbuminuria was not significantly different from the control group. In contrast, individuals with only CBP elevated had a significantly increased risk of both TOD. This suggests that cf-PWV may be a more important driver of CVD than CBP, but inferior to CBP in screening for early manifestations of kidney damage, which may be because cf-PWV is more a measure of large artery stiffness and does not fully reflect the stiffness or function of smaller arteries. However, with the two combined, we found that individuals with both high cf-PWV and high CBP had an even greater risk of microalbuminuria than individuals with elevated CBP alone, which may suggest that the conjoint effect of cf-PWV and CBP on screening for early renal damage is additive.

Multiple stepwise linear regression analysis showed that cSBP was positively significantly associated with CIMT after adjusting for covariates, and age was the main independent influence factor of CIMT, eGFR, and LVMI, while cSBP affected LogACR more compared with age and FBG in our study. As age was a main susceptibility factor of CIMT (30), we stratified age by 60 years and then we found that CIMT abnormality was significantly lower in Group (*CBP*) compared with Group (*control*). A previous study suggested that each SD increase in pPP and cPP was associated with an increased risk of carotid IMT >0.9 mm (31). Various studies have illustrated that cf-PWV was significantly related to CIMT and arterial plaque (3, 32). However, there were few studies conducted to compare associations of CBP with CIMT in a certain level of cfPWV. For the sample size limitation and unclassified CIMT and carotid plaque of our study, further research could be carried out to investigate the relationship between CBP and CIMT in individuals with different degrees of arterial stiffness.

In our study, cAIx@HR75 was significantly associated with LVH [OR = 0.959, *P* = 0.001] after adjusting for covariates. Previous studies showed that pulse pressure amplification (PPA) was tightly associated with LVH (32); higher MAP and central pulse pressure were associated with incident LVH (4). However, opinion on the influence of cAIx@HR75 on LVH varies. In Obayashia's study (33), central AIx was lower in men than in women with hypertension, but the central AIx was not independently associated with the LVMI. It has been shown that in patients with never-treated hypertension, female sex, and shorter height are the important risk factors of elevated radial AIx 75 (34). In our study, cAIx@HR75 was significantly higher in female patients (28.51 ± 10.68 *vs*. 23.43 ± 12.09, *P* < 0.01). However, multiple logistical regression with age stratified by 60 years showed that cAIx@HR75 was not significantly associated with LVH. Aix was associated with arterial stiffness; however, it has been shown that central SBP, but not the central AIx, was an independent determinant of LVH in hypertensive patients in general (33). Further research needs to be conducted to explore the varying relationship of cAIx@HR75 and LVH.

Therefore, each subtype seems to show different associations with certain TOD, and we propose that this is an interesting study direction for further investigation. With larger sample size and prospective studies, by exploring the development trend of diseases in different subtypes of patients, more evidence might be provided for clinical work to guide the focus of screening for TODs and related therapy.

The use of measured systolic and diastolic pressure of brachial artery for calibration of the radial waveform is a conventional method that is applied to the SphygmoCor device and has been widely used. The effect of waveform morphology affecting the relationship between central and peripheral pulse pressure is taken into account by the mathematical transfer function in the device. This accounts for differences in pulse pressure, and the mean pressure is equivalent between central and peripheral locations. However, limitations still remain. Validation of data, as well as the clinical utility of these devices, varies (35); when the radial waveform was calibrated with the oscillometric brachial pressures, the SphygmoCor system could not provide an accurate estimation of central BPs measured invasively. The inherent error in the sphygmomanometric measurement of cuff pressure was the major limiting factor and not the use of the transfer function in clinical settings (36) when compared with invasive values. Recent studies have shown that among different calibration approaches of central aortic blood pressure, including systo-diastolic (SD), calculated mean (CM), and oscillometric mean (OscM) (37–39), CM and OscM were preferred. Further studies, such as validation of a novel method to derive central aortic systolic pressure from the radial pressure waveform using an N-Point moving average method, have been explored as well (13, 40). Hence, the results of this study should be considered in the context of the conventional measurement of BP using a brachial cuff sphygmomanometer when the radial pulse wave is calibrated using brachial SBP and DBP.

Arterial stiffness is influenced by BP, and cfPWV is BP dependent, which has hampered its use in clinical practice. To overcome the limitations, different approaches and parameters have been proposed. The cardio-ankle vascular index (CAVI) is essentially a BP independent index of arterial stiffness, overcoming the limitation of cfPWV. However, CAVI is not equal to the actual, intrinsic stiffness index of the pressure–diameter relationship (β0) but instead varies with BP. A straightforward modification of the formula for calculating CAVI yields a pressure independent version, that is, CAVIo. Actually, PWV in the formula is not equal to cfPWV but PWV measured between the aortic valve and the ankle. Even though some studies have shown that the CAVI formula can equally well be applied to any PWV measurement in pulsatile pressure vessels, including the carotid-femoral PWV (41, 42), we still would expect further studies on the accurate formula of cfPWV. Furthermore, our study calculated CAVIo as a derivation using cfPWV rather than estimating it directly; therefore, we focused on examining the associations of cfPWV with other hemodynamic parameters.

Nevertheless, there are still some limitations in our research. As a cross-sectional study with a small sample size, the results need to be further confirmed in prospective studies. The study was conducted in an Asian population, and it is not known whether the results will hold true for other ethnic groups. To account for possible measurement variations, in future studies consideration could be given to using the average value of the three measurements of cfPWV. In our study, although 10 m/s was chosen as the cutoff of cfPWV based on previous guidelines (7, 43), some limitations still remain. Arterial stiffness was age-related, distributing homogeneously. The age-related reduction in the central-to-periphery stiffness gradient is associated with adverse clinical outcomes (44). For the first time, the study of Bia et al. (42) gave the definition of population-based reference values for PWV, suggesting that age-related stiffness changes were less marked in middle-aged adults and became gradually greater after ~60 years. The majority of the studied population in our study was middle aged, so that cutoff 10 m/s of cfPWV might not adequately characterize the real variations of TOD in different groups in young and older adults. Therefore, additional information might be obtained by accounting for age- and sex-related reference intervals (RIs) for regional and local PWV.

In conclusion, different phenotypes based on the status of cf-PWV and CBP are associated with different TOD. For patients with both elevated cfPWV and CBP, screening for microalbuminuria facilitates the detection of early renal damage. Patients with elevated cfPWV or CBP are recommended to accept echocardiograph Doppler examination for the early detection of risks of LVH. Furthermore, patients with only elevated CBP have an increased risk of ACR abnormality, and central aortic blood pressure evaluation probably helps to assess the risk of early renal impairment. Patients with age over 60 years with both elevated cfPWV and CBP had an increased risk of CKD. The combination of cfPWV and CBP grouping has limited significance for the evaluation of CIMT abnormalities.

## Data Availability Statement

The original contributions presented in the study are included in the article/supplementary material, further inquiries can be directed to the corresponding authors.

## Ethics Statement

The studies involving human participants were reviewed and approved by Ethics Committee of Ruijin Hospital. The patients/participants provided their written informed consent to participate in this study.

## Author Contributions

JZ: conceptualization and funding acquisition. YB, QW, DC, YH, and HC: data curation. YB, QW, BT, and JZ: formal analysis. YB, DC, and JZ: methodology. YB and QW: writing (original draft). AA, BT, and JZ: writing (review and editing). All authors contributed to the article and approved the submitted version.

## Funding

This project was supported by the National Natural Science Foundation of China (Grant No. 81500190), Clinical Science and Shanghai Municipal Hospital New Frontier Technology Joint Project (SHDC12019X20), and Shanghai Municipal Commission of Health and Family Planning (Grant No. 2020YJZX0124).

## Conflict of Interest

The authors declare that the research was conducted in the absence of any commercial or financial relationships that could be construed as a potential conflict of interest.

## Publisher's Note

All claims expressed in this article are solely those of the authors and do not necessarily represent those of their affiliated organizations, or those of the publisher, the editors and the reviewers. Any product that may be evaluated in this article, or claim that may be made by its manufacturer, is not guaranteed or endorsed by the publisher.
